# Uterine Sacculation on Point-of-care Ultrasound in a Pregnant Female Patient: A Case Report

**DOI:** 10.5811/cpcem.2022.2.55216

**Published:** 2022-05-05

**Authors:** Ajay Puri, Hersimran Kaur, Zhanna Roit, Mathew Nelson

**Affiliations:** North Shore University Hospital, Department of Emergency Medicine, Manhasset, New York

**Keywords:** uterine sacculation, point-of-care ultrasound, uterine rupture

## Abstract

**Introduction:**

Uterine rupture is a rare but potentially fatal complication of pregnancy. The incidence of uterine rupture is estimated to be between 0.3 and 11 per 10,000. Additionally, uterine sacculation is a sac or outpouching of the uterus that can lead to uterine rupture in pregnancy. Here we describe a case of a patient who was found to have a uterine sacculation on point-of-care ultrasound in the emergency department (ED) that was complicated by uterine rupture.

**Case Report:**

A 32-year-old female at approximately 18 weeks gestation presented to the ED with three days of abdominal discomfort. The patient’s medical history was significant for prior uterine fibroids requiring recent myomectomy. On arrival the patient was tachycardic, and her abdominal exam revealed distention with mild tenderness to palpation in all quadrants. A point-of-care transabdominal obstetric ultrasound was performed to evaluate the fetal heart rate, which was 157 beats per minute; it also revealed a defect in the uterine wall compatible with a uterine sacculation. The patient underwent magnetic resonance imaging, which revealed a sac-like structure in the fundal portion of the uterus containing a portion of gestational sac and pregnancy contents. Subsequently, she became hypotensive and tachycardic and was taken emergently to the operating room for concern for uterine rupture. Intraoperatively, uterine rupture was confirmed. The patient underwent surgical repair with evacuation of fetal tissue and recovered in the surgical intensive care unit.

**Conclusion:**

Point-of-care ultrasound is a useful and readily available procedure to identify uterine sacculation. Early identification can help escalate the urgency of the patient complaint and may lead to a need for further maternal-fetal evaluation. Emergency physicians should keep a high index of suspicion when evaluating the pregnant patient with a history of uterine surgery.

## INTRODUCTION

Uterine rupture is a rare but potentially fatal complication of pregnancy. The incidence of uterine rupture is estimated to be 11 per 10,000 and 0.3 per 10,000 in women with and without a history of a cesarean delivery, respectively. Additionally, uterine sacculation is a rare pathology that can lead to uterine rupture in pregnancy.^1^ Here we describe a case of a patient who was found to have a uterine sacculation on point-of-care ultrasound (POCUS) in the emergency department (ED) that was complicated by uterine rupture.

## CASE REPORT

A 32-year-old female at approximately 18 weeks gestation by dates presented to the ED with three days of generalized abdominal discomfort, “bloating,” and constipation. The patient endorsed not being able to pass a bowel movement for three days and denied being able to pass flatus on the day of presentation. She denied any episodes of emesis, dysuria, hematuria, vaginal bleeding, or leakage of fluid from her vaginal canal. The patient endorsed feeling normal fetal movements. Her medical history was significant for prior uterine fibroids requiring an abdominal myomectomy in 2019 and a laparoscopic revision of the myomectomy due to persistent fibroids three months prior to conceiving. The patient had a confirmed, single, intrauterine pregnancy on her previous outpatient obstetric visits.

Her vital signs in the ED were as follows: heart rate 111 beats per minute; blood pressure 143/65 millimeters of mercury (mm Hg); oral temperature 98.7° Fahrenheit, respiratory rate of 22 breaths per minute, and an oxygen saturation of 97% on room air. Physical examination revealed a mildly uncomfortable-appearing female with dry mucous membranes. Abdominal exam revealed distention appropriate for 18 weeks gestation with mild tenderness to palpation in all quadrants.

**Image 1:**
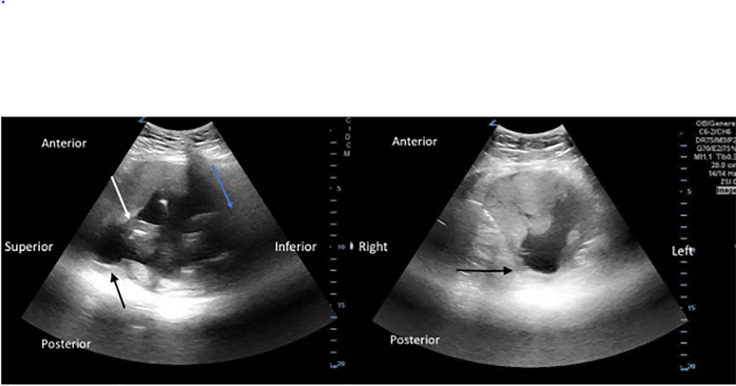
A point-of-care transabdominal obstetric ultrasound in the sagittal and transverse views, respectively. The black arrow demonstrates a defect with thinning of the uterine wall compatible with a uterine sacculation in the uterine fundus. The white arrow indicates fetal parts within the uterus. The blue arrow points toward the inferior portion of the uterus. There was no free fluid surrounding the uterus to suggest uterine rupture at this point.

A 12-lead electrocardiogram revealed her tachycardia to be sinus rhythm. A point-of-care transabdominal obstetric ultrasound was performed to evaluate the fetal heart rate, which was 157 beats per minute, but also revealed a defect in the uterine wall compatible with a uterine sacculation (Image 1).

**Image 2:**
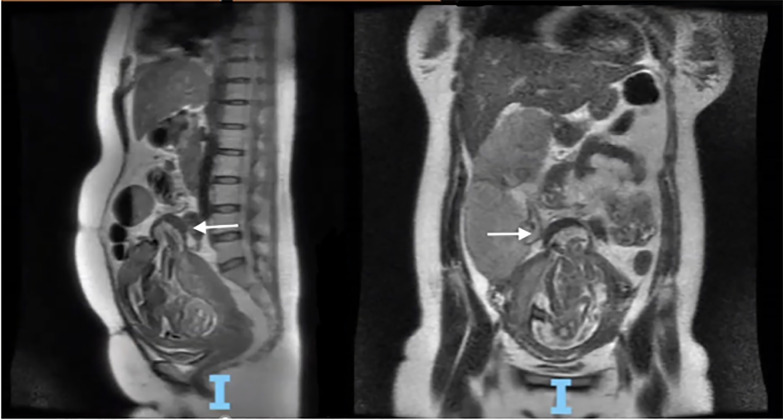
Magnetic resonance imaging with white arrow demonstrating a sac-like structure measuring 4.4 centimeters in the fundal portion of the uterus, containing a portion of gestational sac and pregnancy contents with intact serosa seen in the sagittal and coronal views, respectively.

The obstetrics and gynecology team was emergently consulted given this finding. The consensus was to have the patient undergo an emergent magnetic resonance imaging (MRI) of the abdomen and pelvis to evaluate the sacculation further, as well as to evaluate for a small bowel obstruction, given the patient had been unable to pass bowel movements or flatus and had a history of abdominal surgery. The MRI revealed a sac-like structure measuring 4.4 centimeters (cm) in the fundal portion of the uterus containing a portion of gestational sac and pregnancy contents with intact serosa (Image 2). The radiologist highlighted that the sacculation placed the patient at increased risk of uterine rupture. She was admitted to the obstetrics service to determine next steps in conjunction with the maternal fetal medicine team.

Five hours after admission, the patient began to exhibit increased lethargy, and her skin appeared pale with her blood pressure becoming hypotensive to approximately 80/40 mm Hg and her heart rate becoming tachycardic to approximately 130 beats per minute. She was given two units of emergent-release packed red blood cells and was taken emergently to the operating room for concern for uterine rupture. Intraoperatively, uterine rupture was confirmed with two liters of hemoperitoneum. The patient underwent surgical repair with evacuation of fetal tissue. She recovered well in the surgical intensive care unit.

## DISCUSSION

Uterine sacculation is defined as a transitory pouch or sac-like structure developing from a portion of gravid uterus, containing all layers of the uterus.^1^ Some sources cite the incidence to be 1 in 3000; however, this incidence refers to sacculations in the setting of uterine incarceration. Uterine sacculation has been extensively reported in patients with concomitant uterine incarceration.^2^,^3^ Ultrasound and MRI both are excellent imaging options for uterine sacculation; however, it is likely to be discovered only when a patient is imaged for another differential diagnosis of the presenting complaint.

**CPC-EM Capsule**

What do we already know about this clinical entity?

*Uterine sacculation is a rare outpouching of the uterine wall that has been thought to increase the chances of uterine rupture*.

What makes this presentation of disease reportable?

*Our patient was correctly identified to have a uterine sacculation on a point-of-care ultrasound (POCUS), which expedited emergency department management*.

What is the major learning point?

*Uterine sacculation can progress to uterine rupture in a short time. It can be identified on POCUS and warrants emergent obstetrics consultation*.

How might this improve emergency medicine practice?

*Adding uterine sacculation to the list of differential diagnoses and knowing that it may be identified on POCUS will improve care of the pregnant patient*.

Risk factors for uterine sacculation include uterine malformations, endometriosis, a primary myometrial defect, and prior surgery. In contrast, our patient had a sacculation containing fetal parts through a portion of the uterine fundus likely weakened by her prior laparoscopic myomectomy. It is unclear how often the precise process that occurred in our patient happens. On POCUS, our patient presented with a defect in the uterine wall with concomitant thinning of the wall compared to the rest of the uterus. Given the corroboration on MRI, such findings should raise suspicion of a uterine sacculation with possible impending rupture. Uterine rupture is suggested by free fluid surrounding the uterus in the setting of abdominal pain, distention, or hemodynamic instability.

Most uterine ruptures are associated with patients who have had a prior cesarean section and are attempting a trial of labor.^4^ Upon review of the literature, we found that 31% of cases of uterine rupture occurred in women with prior surgery involving the uterus with 36% of these cases following a laparoscopic myomectomy, which our patient had previously received.^5^ The most frequently encountered symptom or sign prior to diagnosis of uterine rupture is a fetal heart rate abnormality, usually fetal bradycardia, occurring in up to 87.5% of cases.^4^ This is followed by abdominal pain, vaginal bleeding, altered uterine contractions, and hypotension.^6^ In our case, our patient’s abdominal discomfort and tenderness to palpation is what eventually led to her diagnosis of uterine sacculation, but she did not have any identified fetal heart rate abnormalities. Determining a fetal heart rate, even in the fetus that is not yet viable, may be clinically important as it may allude to critical illness affecting the patient.

Our patient’s pregnancy was deemed high risk by her obstetrician as she conceived five months after her laparoscopic myomectomy, despite a six-month minimum recommendation. To our knowledge, sources differ on the recommended time period and there isn’t clear literature to support the time periods recommended. Some have suggested three to six months, others recommend waiting at least six months to avoid uterine rupture.^7^,^8^ A case series of 14 uterine ruptures after laparoscopic myomectomy by Kim et al found the time interval between uterine rupture and myomectomy was between 12–84 months, highlighting that the risk of uterine rupture persists even after significant time has passed since myomectomy.^9^ Hence, it is important to determine whether the pregnant patient presenting with abdominal complaints has had a history of uterine surgery, particularly with recent proximity to conception.

## CONCLUSION

Uterine rupture is a devastating intrapartum emergency. It may be preceded by a uterine sacculation caused by prior uterine surgery. Point-of-care ultrasound is a useful and readily available procedure to identify this rare pathology. Early identification can help escalate the concern of our obstetric colleagues and determine the need for further maternal fetal evaluation. Emergency physicians should keep a high index of suspicion when evaluating the pregnant patient with a history of uterine surgery even without the late clinical findings of fetal bradycardia, severe abdominal pain, or heavy vaginal bleeding.
